# Functional mechanisms of drought tolerance in subtropical maize (*Zea mays* L.) identified using genome-wide association mapping

**DOI:** 10.1186/1471-2164-15-1182

**Published:** 2014-12-24

**Authors:** Nepolean Thirunavukkarasu, Firoz Hossain, Kanika Arora, Rinku Sharma, Kaliyugam Shiriga, Swati Mittal, Sweta Mohan, Pottekatt Mohanlal Namratha, Sreelatha Dogga, Tikka Shobha Rani, Sumalini Katragadda, Abhishek Rathore, Trushar Shah, Trilochan Mohapatra, Hari Shankar Gupta

**Affiliations:** Division of Genetics, Indian Agricultural Research Institute, Pusa, New Delhi, 110012 India; Maize Research Centre, ARI, Acharya N G Ranga Agricultural University, Rajendra Nagar, Hyderabad, 500030 India; Agricultural Research Station, Acharya N G Ranga Agricultural University, Karimnagar, 505001 India; International Crops Research Institute for the Semi-Arid Tropics, Patancheru, 502324 India; National Research Centre on Plant Biotechnology, Pusa, New Delhi, 110012 India

**Keywords:** Genome-wide SNPs, Association mapping, Functional mechanisms, Candidate SNPs, Water stress, Drought tolerance, Maize

## Abstract

**Background:**

Earlier studies were focused on the genetics of temperate and tropical maize under drought. We identified genetic loci and their association with functional mechanisms in 240 accessions of subtropical maize using a high-density marker set under water stress.

**Results:**

Out of 61 significant SNPs (11 were false-discovery-rate-corrected associations), identified across agronomic traits, models, and locations by subjecting the accessions to water stress at flowering stage, 48% were associated with drought-tolerant genes. Maize gene models revealed that SNPs mapped for agronomic traits were in fact associated with number of functional traits as follows: stomatal closure, 28; flowering, 15; root development, 5; detoxification, 4; and reduced water potential, 2. Interactions of these SNPS through the functional traits could lead to drought tolerance. The SNPs associated with ABA-dependent signalling pathways played a major role in the plant’s response to stress by regulating a series of functions including flowering, root development, auxin metabolism, guard cell functions, and scavenging reactive oxygen species (ROS). ABA signalling genes regulate flowering through epigenetic changes in stress-responsive genes. ROS generated by ABA signalling are reduced by the interplay between ethylene, ABA, and detoxification signalling transductions. Integration of ABA-signalling genes with auxin-inducible genes regulates root development which in turn, maintains the water balance by regulating electrochemical gradient in plant.

**Conclusions:**

Several genes are directly or indirectly involved in the functioning of agronomic traits related to water stress. Genes involved in these crucial biological functions interacted significantly in order to maintain the primary as well as exclusive functions related to coping with water stress. SNPs associated with drought-tolerant genes involved in strategic biological functions will be useful to understand the mechanisms of drought tolerance in subtropical maize.

**Electronic supplementary material:**

The online version of this article (doi:10.1186/1471-2164-15-1182) contains supplementary material, which is available to authorized users.

## Background

Drought at the flowering stage produces infertile pollen in maize (*Zea mays* L.) [1], resulting in substantially lower yield. A shorter anthesis-to-silking interval (ASI) was observed under drought in a lowland tropical maize population [2], and the interval was considered to be an indirect selection criterion for grain yield [3]. Grain yield, a complex trait dependent on several environmental factors [4], was increased through genetic modifications despite stress [5].

Several researchers have identified drought-related genes in different tissues that regulate molecular and physiological responses under stress [6–8]. In the ears and silks of maize, some genomic regions that control the levels of abscisic acid (ABA) and sugar showed the signal transduction involved in stress-related pathways and regulated kernel size and productivity of the plant under drought [6]. Different tissues showed the expression of an NAC-transcription-factor-encoding gene [9], which was observed to be strongly associated with the relative ear position, 100-kernel weight, and flowering time across the experimental maize population [10]. Some drought response has also been recorded recently in reproductive and leaf meristem tissues in maize [7].

Furthermore, a high-resolution association map created using a high-density marker set has the potential to unravel stress-associated genetic variability in a genome [10]. Considerable efforts have been made to understand the association of SNPs with different phenotypic traits in maize [10–12], and strong SNP associations have been identified for flowering time [13], kernel shape [14], 100-kernel weight [14], and kernel quality [15]. Moreover, target genes for crop improvement have been successfully identified using genome-wide association (GWA) mapping in maize [6, 10, 12].

In this study, we assembled a GWA mapping panel using elite subtropical maize genotypes and phenotyped it under water stress (WS) at flowering time at multiple locations to identify the SNPs associated with key agronomic traits. The association of genetic loci with the agronomic traits and their recurrence level across different locations and models were examined, to identify the SNPs significantly associated with the candidate genes that interact to confer tolerance to WS.

## Results

### Phenotypic data

An association mapping panel of 240 subtropical maize lines was subjected to WS and the performance of the stressed maize lines was compared with that of the lines grown under a well-watered (WW) environment. The panel was phenotyped for anthesis-to-silking interval (ASI), ear length (EL), ear girth (EG), kernels per row (KR), the number of kernel rows (KRN), 100-kernel weight (HKW), and grain yield (GY) at three locations: Indian Agriculture Research Institute (IARI), New Delhi; Acharya N G Ranga Agricultural University (ANGRAU), Hyderabad; and Agricultural Research Station (ARS), Karimnagar for two years. The overall performance of the hybrids under WS was significantly impaired [Additional file 1: Table S1]. The phenotypic correlation coefficient showed that GY and the traits contributing to it were positively and significantly correlated with each other, and ASI was negatively but significantly correlated with other agronomic traits [Additional file 2: Table S2].

### Genome-wide association mapping

#### Genome-wide association models

**Figure 1:**
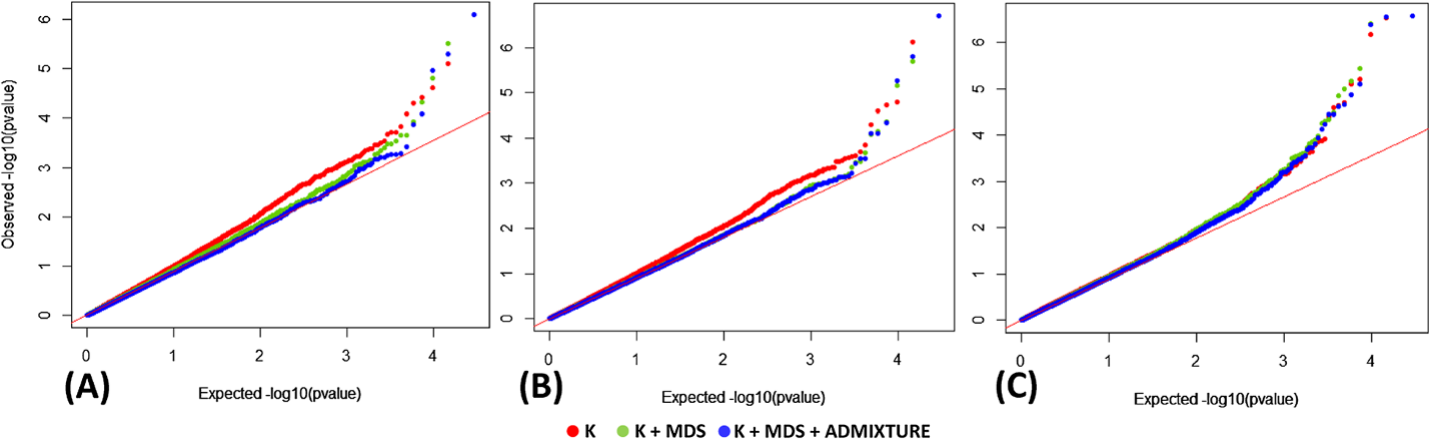
**QQ plots showing the effect of higher models on correcting population structure.** QQ plots showing the ratio of the observed and the expected *P* values for **(A)** ASI–Hyderabad, **(B)** ASI–Karimnagar, and **(C)** GY–Hyderabad. The correction of kinship varied among the three GWA models: K, Q_10_ + K (M1), and Q_10_ + K + Admixture (M2). The QQ plots were determined by means of GenABEL models using a set of 29 619 SNPs.

We detected GWA signals from GenABEL and Genome Association and Prediction Integrated Tool (GAPIT) models. Model fitness varied over the data sets (traits + models + locations) and an example from GenABEL is given below. For ASI–Hyderabad, a higher model (Q_10_ + K + Admixture) from GenABEL showed greater fitness than the lower hierarchy models (K and Q_10_ + K) did. The genomic inflation factor (λ) was 1.04 in the latter model but was reduced to 1.02 when admixture was added as a covariate (Figure 1a); Q_10_ + K and Q_10_ + K + Admixture from GenABEL had equal effect on correcting cryptic relationship for ASI–Karimnagar (Figure 1b); and all the models proved equally good for GY–Hyderabad (Figure 1c).

#### Comparison of GWA signals: WW and WS

Of 61 SNPs strongly (*P* < 5.8 × 10^−4^) associated with WS, 11 were false-discovery-rate (FDR)-corrected associations and the rest (50) were consistent across the data sets (traits + models + locations). The maximum number (16) of association signals were identified on chromosome 5 followed by those on chromosome 3 (15), whereas chromosomes 2 and 8 shared the least number of significant associations (2) across traits measured under WS.

Genome-wide analysis was also assessed for the WW data sets, which mapped 70 significant associations (*P* < 5.8 × 10^−4^) including 9 FDR-corrected associations. The maximum number of GWA signals for WW was contributed by the SNPs mapped on chromosome 10 (15) followed by those on chromosomes 1 (12) and 7 (10), whereas the minimum number (2) of GWA signals was shared by the SNPs mapped on chromosome 8. The recurrence of these signals across all data sets (traits + models + locations) varied with those observed in WS. Also the stringency of these results was observed through the number of significantly associated SNPs for EG trait being 17 in WS and 24 in WW. But, for traits ASI, GY, and KR, the number of GWA signals in WW were equivalent to that in WS. SNPs significantly associated in both conditions were mapped near both drought tolerant genes and regulatory genes having their importance in both stressed and normal environment–PZE-107021672 and PZE-107021673 for GY; PZE-101135368 for ASI and KR; PZE-107110985 for ASI, GY, and KR. However rest of the SNPs were also significantly associated in WW and WS but were specific to environmental locations.

#### GWA signals: WS

**Figure 2:**
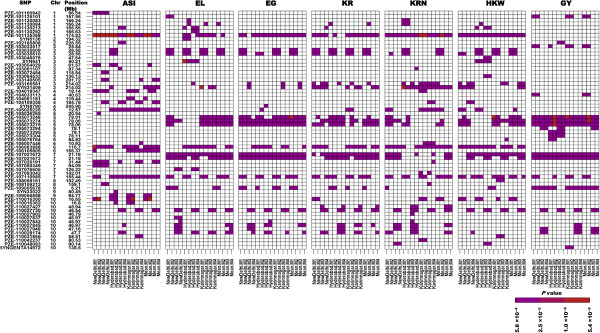
**Significant associations (** ***P*** **< 5.8 × 10** ^**−4**^ **) under water stress observed across agronomic traits and locations.** All 64 strong associations for WS (*P* < 5.8 × 10^−4^ as the cut-off) that were observed on 10 maize chromosomes were pooled over a heat map. The strongly associated SNPs are appended by their positions on the chromosomes. Based on the *P* values, a heat map was generated for seven agronomic traits – anthesis-to-silking interval (ASI), ear girth (EG), ear length (EL), grain yield (GY), 100-kernel weight (HKW), kernels per row (KR), and the number of kernel rows (KRN) – recorded at three locations and mean of phenotypic data at all locations, and four models (see Methods for details) under waters-stressed conditions. Out of these 64 SNP associations, 31 were close to the drought-tolerant genes that regulate some molecular and physiological functions leading to drought tolerance (refer Figure 4).

On chromosome 1, a significant signal (PZE-101135368) associated with WS-ASI was located 12 kb from the nuclear factor-YA transcription factor (NF-YA) [Additional file 3: Table S3]. This SNP also showed an FDR-corrected *P* value of 5.4 × 10^−9^ for WS-ASI and was repeated 75% of the times across all data sets (traits + models + locations) under WS (Figure 2). For ASI–New Delhi, PZE-101135368 was significantly associated (at 5% FDR) with a stronger *P* value of 5.4 × 10^−9^ for WS [Additional file 3: Table S3] than that for the non-significant signal observed under WW (*P* = 1.4 × 10^−4^) [Additional file 4: Figure S1]. In addition, this locus was co-localized with a quantitative trait locus (QTL) earlier mapped for ASI by Almeida *et al* [16]. Furthermore, significant associations for WS also comprised DnaJ-49-like chaperone protein and a domain of unknown function (231) that were co-localized with two other QTLs for ASI on chromosome 1 under WS [16].

**Figure 3:**
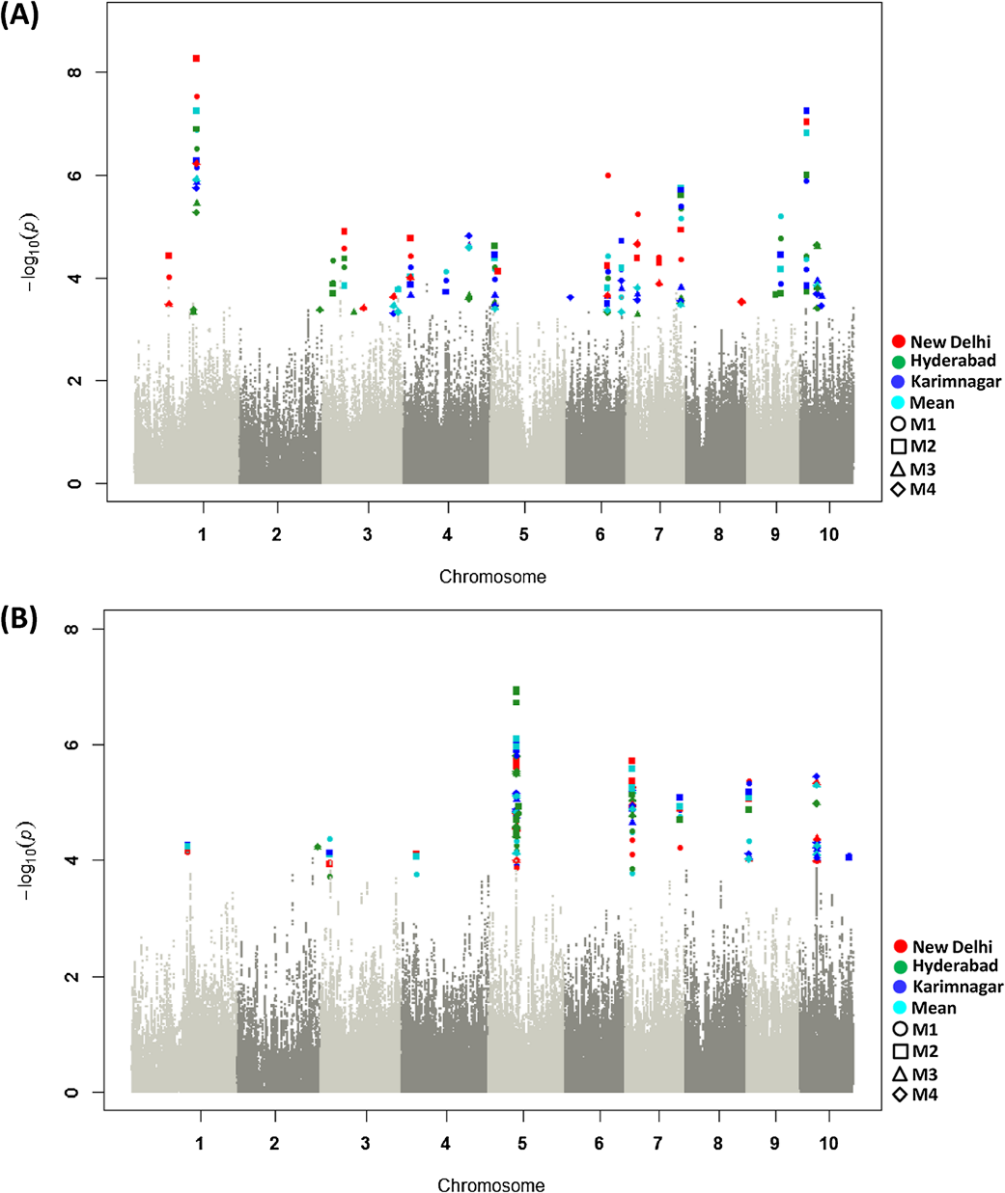
**SNPs significantly associated for anthesis-to-silking interval and grain yield across all locations and models under water stress.** All log_10_ *P* values (<−4.0) observed for a data set were pooled over a GWA plot. **(A)** Six FDR-corrected associations on 5 chromosomes were observed for WS-ASI specific to locations and models. PZE-101135368 on chromosome 1 demonstrated strong signals at all locations and models, the most significant (*P* = 5.4 × 10^–9^) bring for the data set New Delhi–M2. **(B)** Eight FDR-corrected associations were observed for WS-GY. Four of these were mapped on chromosome 5, with the PZE-105073248 and PZE-105073275 SNPs showing strong association across all data sets.

Two FDR-corrected associations for WS-ASI, one on chromosome 7 (PZE-107110985) and the other on chromosome 10 (PZE-110016300), were found near a gene encoding a basic helix-loop-helix (bHLH) transcription factor [Additional file 3: Table S3]. The first locus identified at 92 kb from bHLH was also significantly associated with WS-GY, -EG, -HKW, -EL, -KRN, and -KR at specific locations [Additional file 3: Table S3]. The second locus, mapped at 118 kb from bHLH, also showed strong associations with WS-HKW, -KRN, and -KR [Additional file 3: Table S3] and significant associations for ASI across all locations and mean data (Figures 2 and 3a), while the rest of the traits were confined to specific locations (Figure 2).

SNP PZE-103149505 mapped on chromosome 3, indicated strong GWA signals for WS-ASI across multiple locations (Figure 2). These signals showed a stronger and significant association under WS than that for WW, in which the *P* values were less significant and less repeatable. This SNP was mapped 30 kb from a stress-related gene encoding NADP-malic enzyme [Additional file 3: Table S3].

SNPs mapped on chromosome 5 showed five FDR-corrected association signals for WS-GY [Additional file 3: Table S3]: three SNPs (PZE-105073248, PZE-105073274, and PZE-105073275) were consistent across all locations, whereas two (PZE-105073295 and PZE-105076764) were specific to the locations and models (Figure 3b). Of these five SNPs, three (PZE-105073248, PZE-105073275, and PZE-105073274) were clustered in a 131 kb region, which enclosed genes encoding MYB-related (MYB) transcription factor and squamous promoter binding protein (SBP) transcription factor [Additional file 3: Table S3]. This cluster was identified 65% of the times across all WS data sets, including GY (Figure 2), and showed strong associations with WS-GY that were more recurrent across locations and models than those in the WW data sets. In addition to this, two FDR-corrected SNPs (PZE-107021673 and PZE-107110985) on chromosome 7 were also strongly associated with WS-GY. The first SNP was mapped nearer to the *glycerol-3-phosphate dehydrogenase* gene and an ethylene responsive factor (ERF) transcription factor, and the second was close to the bHLH encoding gene [Additional file 3: Table S3]. Another SNP on chromosome 9, 16 kb from the MYB, was also associated with WS-GY [Additional file 3: Table S3]. All SNPs on chromosomes 7 and 9 also had a significant association with WS-EG [Additional file 3: Table S3].

Two SNPs on chromosome 5 (PZE-105025225 and PZE-105076764) mapped close to a gene encoding a C2H2-type zinc finger (C2H2) transcription factor were independently associated for WS-ASI and -GY when measured at Hyderabad. The first SNP (PZE-105025225), 31 kb from C2H2, showed a strong association (*P* = 2.4 × 10^−5^) for WS-ASI. The second SNP (PZE-105076764), 7 kb from C2H2, was associated with a strong signal (*P* = 1.2 × 10^−5^) for WS-GY [Additional file 3: Table S3], which was non-significant under WW. Also, SNPs mapped on chromosomes 2 (PZE-102185808) and 7 (PZE-107110985) were associated with both WS-ASI and -GY (Figure 3a, b) where the association of the first locus with ASI was more significant in WS than that in non-significant signals observed under WW at Hyderabad. The first locus was mapped near the genes encoding MYB, a bZIP transcription factor, and a zinc finger-homeodomain (ZF-HD) transcription factor, and the second was near bHLH [Additional file 3: Table S3].

For WS-HKW, four strong GWA signals were observed on different chromosomes close to the stress-related genes. The first two loci (PZE-101135368 on chromosome 1 and PZE-105025225 on chromosome 5) were observed 12 kb and 20 kb from NF-YA respectively [Additional file 3: Table S3]. The third locus (PZE-106063888 on chromosome 6), also showing a significant association for WS-HKW (*P* = 6.4 × 10^−5^), was mapped close to one gene, namely *vacuolar proton ATPase pump* (*V-type H*^*+*^*pump*), and two transcription factors, namely NAC and calmodulin-binding transcription activator (CAMTA) [Additional file 3: Table S3]. The fourth locus (PZE-103035609 mapped on chromosome 3) was close to a universal stress protein [Additional file 3: Table S3].

## Discussion

### Metabolic mechanisms common to WS and WW conditions

**Figure 4:**
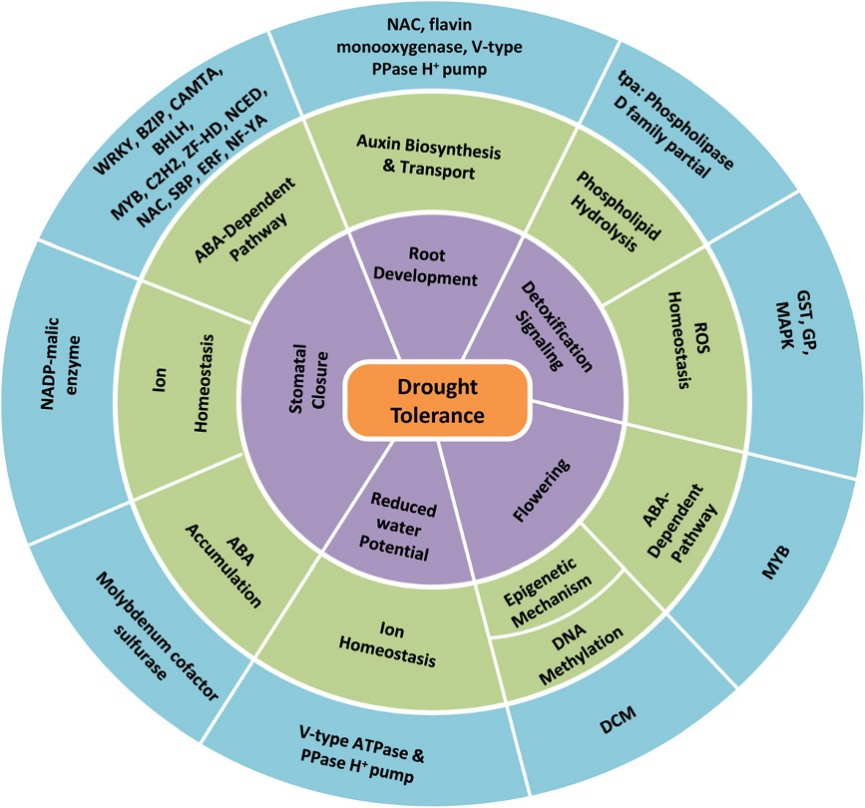
**Functional roles of candidate genes associated with drought tolerance.** The outermost circle represents the drought-tolerant genes mapped in the present study; the middle circle shows the molecular mechanisms involved in drought tolerance; and the inner circle describes the functional role of the GWA candidate genes that directly lead to drought tolerance.

Strong GWA signals were detected near the genes responsible for drought tolerance as well as for plant metabolism across both WS and WW environments. This commonality highlights the importance of genes that are responsible for maintaining yield and were associated with yield-related traits under both the environments. Two SNPs (PZE-107021672 and PZE-107021673) on chromosome 7 were strongly associated with GY for both WS (Figure 2) and WW data sets [Additional file 4: Figure S1, Additional file 5: Figure S2, Additional file 6: Figure S3, and Additional file 7: Figure S4]. These SNPs showed a drought-tolerant ERF transcription factor in their vicinity, which promotes ABA-dependent stomatal closure under WS [17] (Figure 4). Apart from this drought-tolerant gene, these SNPs also included two more genes encoding MADS-box and glycerol-3-phosphate dehydrogenase (G3PDH), which regulate generalized functions in plants. During root development, the MADS-box gene regulates auxin transport [18], cell proliferation [19], the transition to flowering and flower development [19]. G3PDH is part of the mitochondrial glycerol-3-phosphate shuttle system that is induced at different stages of plant development when the demand for redox adjustment is high [20]. G3PDH is also one of the enzymes involved in glycerol metabolism, where the role of glycerol in regulating root development through multiple pathways in *Arabidopsis* has been studied recently [21].

A strong GWA signal (PZE-101135368) for ASI and KRN was identified for both WS [Additional file 3: Table S3] and WW across all data sets although the *P* value varied with the data set [Additional file 4: Figure S1, Additional file 5: Figure S2, Additional file 6: Figure S3, and Additional file 7: Figure S4]. This association pointed to a drought-tolerant NF-YA transcription factor 12 kb from the SNP. Under WS, NF-YA promotes ABA-dependent stomatal closure, which contributes to drought tolerance [22] (Figure 4). NF-YA is also important to several other functions including male gametogenesis, embryogenesis, and seed development under WW conditions [23]. The consistency of these GWA signals shows that NF-YA is important to high yields irrespective of whether if water is scarce or abundant.

Another strong GWA signal (PZE-107110985) was that for ASI, GY, and KR across both environments and at all locations. *BHLH*, a drought-tolerant gene, was mapped close to this SNP, and has been known to interact with two other two drought-tolerant genes, *bZIP* and *MYB*, which are responsible for tissue-specific flavonoids production and light responsiveness under normal conditions [24]: *bZIP* regulates auxin transport [25] and *MYB* promotes light signalling transductions including photosynthesis in *Arabidopsis thaliana*. The importance of these genes under WS was noticed from the specific GWA signals for WS that were mapped close to three drought-tolerant gene families: PZE-103061107 near *MYB*, PZE-104061181 and SYNGENTA14972 near *bHLH*, and PZE-104109358 near *bZIP*. Apart from drought tolerance, these genes can also interact strongly to regulate other generalized functions, as can be inferred from the GWA signals mapped across both the environments.

### Mechanisms of drought tolerance

Strong GWA signals were recorded near the interacting drought-tolerant genes under WS [Additional file 3: Table S3] (Figure 4). These mechanisms, namely stomatal closure, detoxification signalling, root development, reduced water potential, and flowering, are influenced at different physiological and functional levels and enhance drought tolerance (Figure 4).

#### Stomatal closure

**Figure 5:**
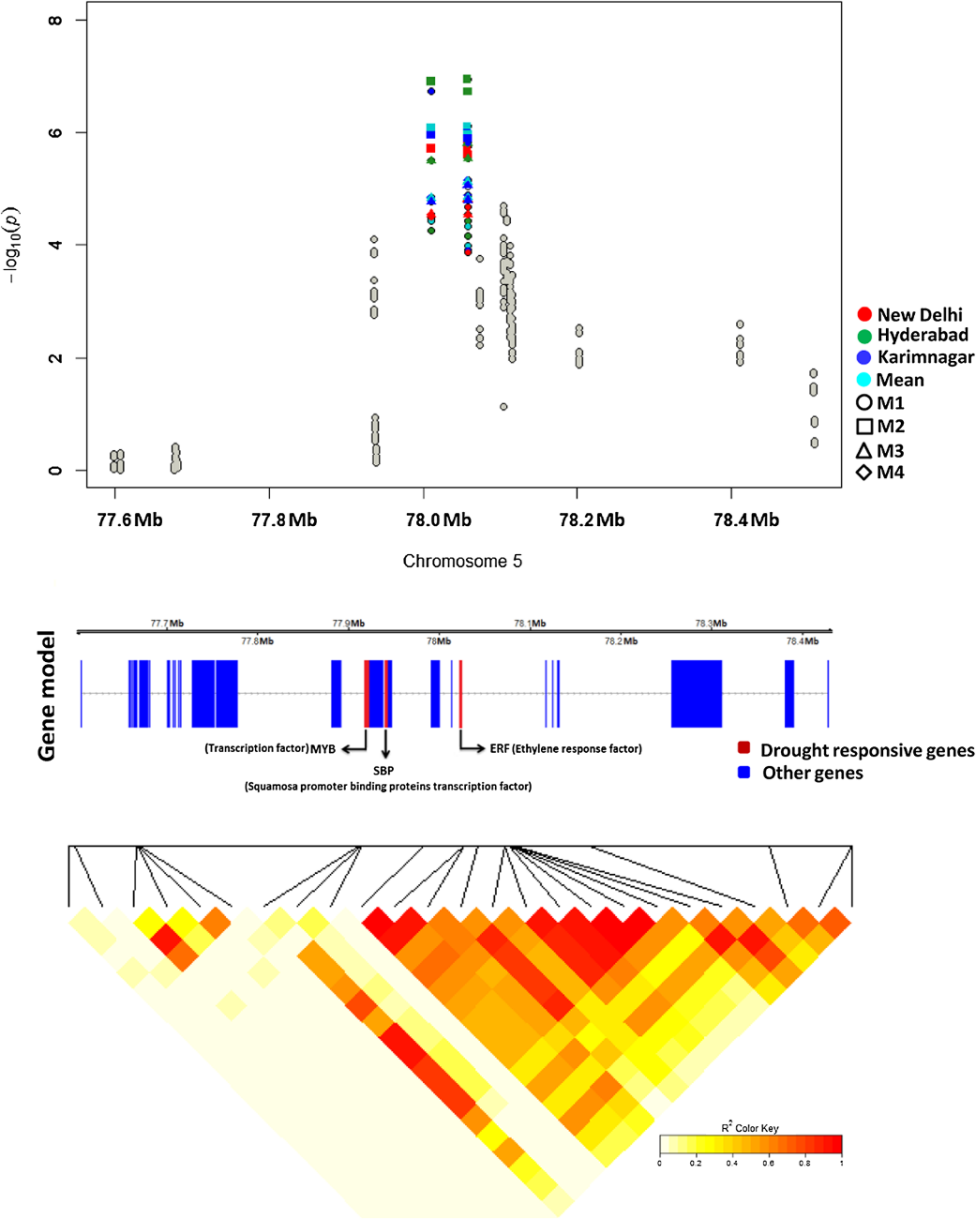
**Regional association plot of 1 Mb window on chromosome 5 showing SNPs significantly associated for grain yield under water stress across all locations and models.** The genomic region included three neighbouring SNPs (PZE-105073248, PZE-105073274, and PZE-105073275) that were strongly associated with grain yield. PZE-105073274 and PZE-105073275 (78.05 Mb; 253-bp apart) in high linkage disequilibrium (*r* ^2^ = 1) showed FDR-corrected associations specific to locations and models. All three SNPs were observed close to *MYB* and *SBP*: 84 kb, 130.8 kb, and 131.1 kb from *MYB* and 65 kb, 111.6 kb, and 111.8 kb from *SBP* for PZE-105073248, PZE-105073274, and PZE-105073275 respectively.

A genomic region encoding two neighbouring SNPs 253 bp apart (PZE-105073274 and PZE-105073275) in high linkage disequilibrium (LD) (*r*^2^ = 1) [26] was seen on chromosome 5, near two CAMTA regulating factors, the MYB and SBP (Figure 5). These SNPs showed significant *P* values for several traits including GY, EG, EL, and KR under WS [Additional file 3: Table S3]. The extended regions with strong LD exhibited a steep selection and genetic drift [27] of those traits in the population. *CAMTA* was also identified near a locus mapped on chromosome 6 that was strongly associated with WS-ASI, -EG, -EL, -KRN, -KR, and -HKW [Additional file 3: Table S3]. CAMTA (at 115 Mb on chromosome 6), MYB, and SBP (at 77.9 Mb on chromosome 5) contributed to stomatal closure, which promotes drought tolerance, presumably through strong inter-chromosomal LD. Other CAMTA-regulating factors such as WRKY and bZIP were mapped near an SNP (PZE-104109358) on chromosome 4 that was strongly associated with WS-ASI, -KRN, and -HKW [Additional file 3: Table S3].

An SNP cluster on chromosome 5 with a significant association for WS-GY and -HKW included the genes encoding C2H2 and MYB [Additional file 3: Table S3]. Although the function of *C2H2* in maize is not clear, a *C2H2*-type zinc finger was shown to play a defensive role in oxidative stress generated under different forms of environmental stress in *Arabidopsis* [28]. Moreover, a gene of the *MYB* family is known to integrate ABA and auxin signalling under WS [29].

In the present study, two SNPs were identified, one on chromosome 3 and one on chromosome 4, with strong associations for WS-ASI and WS-KRN [Additional file 3: Table S3] (Figure 2). The SNP on chromosome 3 encompassed the gene encoding 9-cis-epoxycarotenoid dioxygenase (NCED) whereas the one on chromosome 4 was localized at a point close to a molybdenum cofactor sulfurase encoding gene [Additional file 3: Table S3]. Because of possible inter-chromosomal LD, this region may contribute to ABA-dependent stomatal closure. This defensive mechanism can be explained by an integrative ABA synthesis, accumulation [30], and signalling in guard cells [31]. We also identified two SNPs, one on chromosome 3 and another on chromosome 10, close to an NADP-malic enzyme encoding gene [Additional file 3: Table S3], with strong *P* values for WS-ASI and -GY, respectively (Figure 3a, b). This gene promotes stomatal closure, which induces tolerance during WS, by removing malate from guard cells [32].

#### Detoxification signalling

Detoxification signalling is a major response to environmental forms of stress, and WS is known to stimulate oxidative stress in chloroplasts, mitochondria, and peroxisomes [33], thereby leading to the production of reactive oxygen species (ROS) that are sensitive to ABA-signalling pathways [33] and induce programmed cell death in plants [34]. Therefore, removal of free oxygen radicals may represent the primary defence mechanism in drought tolerance. Glutathione-*S*-transferase (GST) and glutathione peroxidase (GP), which are responsible for ROS homeostasis, change turgor pressure through an ABA-mediated pathway [35, 36] in order to sustain the plant under drought. These genes were observed to be strongly associated with WS-GY, -HKW, and -KRN [Additional file 3: Table S3], thereby highlighting the role of ROS homeostasis under WS (Figure 4).

Phospholipid hydrolysis is also known to play a role in detoxification signalling under WS [37, 38]. In the present study, a genomic region (chromosome 3) encoding phospholipase D (PLD) displayed a strong and WS-specific association signal for GY [Additional file 3: Table S3]. PLD is a signalling messenger that facilitates stomatal closure, cell viability, and enhanced root growth during stress [38].

#### Root development

Under WS, auxin signalling results in a deeper root system, which increases water use efficiency [39, 40]. We noticed two strong association signals in the genomic regions that included the genes for auxin transport (*NAC*) and auxin biosynthesis (*flavin monooxygenase*) on chromosomes 6 and 10 respectively [Additional file 3: Table S3]. These regions showed high *P* values for WS-ASI, -KR, and -HKW, suggesting that maintenance of the root system was in fact associated with flowering and seed development stages.

#### Reduced water potential

Vacuolar proton pumps reduce water potential under WS: their expression in the root system enhances water-absorbing efficiency [41]. The strong signal of the *vacuolar proton pyrophosphatase pump* (*V-type PPase H*^*+*^*pump*) for WS-KRN and that of the *V-type ATPase H*^*+*^*pump* for WS-ASI, -EG, -HKW, -EL, -KRN, and -KR [Additional file 3: Table S3] establish a proton electrochemical gradient across the vacuolar membrane [41, 42], thereby enhancing ion homeostasis, which is responsible for maintaining osmotic balance and in turn, coping with drought (Figure 4).

#### Flowering

Drought tolerance also involves changes in flowering through epigenetic mechanisms and the ABA-dependent pathway – an observation borne out by the present study (Figure 4) –and DNA cytosine methylation and histone modifications (ABA levels are regulated by histone) are innate to epigenomes [43, 44]. Our study identified 15 significant SNPs [Additional file 3: Table S3] close to genes encoding DNA-cytosine-5-methyltransferase (DCM) and MYB transcription factor that are involved in epigenetic mechanisms and ABA signalling pathways under WS, thereby pointing to their role in drought tolerance.

#### Functional relationship of stress-responsive genes

Interactions of several functional mechanisms associated with drought tolerance are discussed below. Epigenetic changes such as DNA methylation respond differently at tissue and developmental levels. In a study of the association of methylation-specific sites with drought tolerance, methylation was reduced in response to water stress at the tillering stage whereas drought-induced DNA methylation and demethylation were higher at the tillering stage than at the booting and heading stages [45]. Epigenetic changes in *MYB*, an ABA-signalling transcription factor, have been found to be specific to tissues including pericarp and cob in maize [46]. These epigenetic changes include site-specific methylation patterns, which have been studied in ABA-signalling genes including the *MYB* gene family, where stomatal development genes such as *MYB44* are demethylated and expressed in response to WS [47]. MYB proteins also promote stamen development and ensure adaptation to WS [48]. Therefore the epigenetic changes in ABA-signalling genes can possibly lead to changes in flowering under WS (Figure 4).

ROS are produced in an ABA-signalling pathway in which two ABA-signalling transcription factors C2H2 and MAPK are negatively regulated by *ERF* [49], which is repressed under WS [50]. ROS are scavenged by a detoxification signalling process [37], in which PLD is reported to influence ABA responses and opening and closing of stomata through bifurcating pathways [51]. When PLD activity was studied under water stress, three ABA-signalling genes, namely *MYB*, *NAC* (PZE-108058161; chromosome 8), and *bHLH*, showed differential regulation in both wild-type and antisense-PLD *Arabidopsis* [52]. Therefore, networking among ABA and PLD signals is one of the important defensive mechanisms against WS. This suggests that the ROS generated by ABA signalling are reduced by the interplay between ethylene signalling, ABA signalling, and detoxification signalling under WS.

Guard cell signal transductions regulate CO_2_ influx for photosynthesis as well as water loss through stomatal closure under WS [31, 32]: ABA signalling promotes the closing of stomata, which reduces CO_2_ intake in guard cells and thereby decreases the rate of photosynthesis. To maintain the rate of photosynthesis under WS, plants adopt an alternative pathway for delivering CO_2_. This alternative pathway includes malate degradation, catalyzed by a respiratory enzyme, NADP-malic enzyme, which releases CO_2_ as a secondary source for CO_2_ influx under WS [32].

ABA is a hormone released in response to various forms of abiotic stress and regulates tolerance through a network of interconnected genes. The mechanisms of drought tolerance are stimulated by both ABA-dependent and ABA-independent pathways: C2H2, for instance, is part of an ABA-dependent pathway as well as that of an ABA-independent pathway. C2H2 zinc finger proteins with ERF-associated amphiphilic repression (EAR) motifs have been reported to be responsive to ABA [53] and to WS [54].These findings suggest that ABA and ethylene interact under drought and enhance tolerance by promoting stomatal closure. Under WS, *C2H2* acts as a transcriptional regulator through an ABA-independent pathway [55]: ABA-independent C2H2 triggers the genes that are related to H_2_O_2_ homeostasis and thus decreases the amount of H_2_O_2_ in guard cells [56]. However, stomatal closure as part of the drought response is promoted by ABA and H_2_O_2_ accumulation in guard cells [56].

The electrochemical gradient generated by the proton pump (*V-type PPase H*^*+*^*pump* mapped near PZE-110027802; chromosome 10) promotes the secondary active transport of sugar molecules (*hexokinase 3* mapped near PZE-108106212; chromosome 8) into the vacuole. This helps the plant in maintaining its internal water balance [57]. In addition, the proton pump has also been reported in roots [42], which suggest that this pump regulates water balance in roots and thus contributes to drought tolerance. It is well understood that the water balance in plants is interrelated to root development, where the auxin-inducible genes that promote root development are regulated by the *C2H2* [58]. This suggests that the integration of root development and ABA signalling molecule C2H2 allows the maintenance of an electrochemical gradient in the plant.

## Conclusions

Genome-wide association analysis, using multiple locations and two models, identified SNPs from agronomic traits linked with the genes directly or indirectly associated with drought tolerance in subtropical maize lines. These genes uncovered physiological responses and molecular mechanisms related to drought tolerance. Genes governing several functional traits were identified, including stomatal closure, reduced water potential, root development, signalling pathways, and flowering. These genes interact extensively to help the plant cope with drought. SNPs and their functional association with several drought-responsive genes will be useful in elucidating the mechanism of drought tolerance in subtropical maize.

## Methods

### Genetic material

Our experiment was based on a panel of 240 elite inbred lines of subtropical maize [26] from several Indian breeding programmes and also included genotypes from CIMMYT, Mexico adapted to subtropical climates. The lines were separated into three groups, namely early, medium, and late, based on the number of days to flowering.

### Phenotyping

Field experiments were laid out in an alpha-lattice design at three locations: IARI, New Delhi (28°N 77°E; 229 Amsl); Maize Research Centre, Acharya N G Ranga Agricultural University, Hyderabad (17°N 78°E; 536 Amsl), and ARS, Acharya N G Ranga Agricultural University, Karimnagar (18° N 79°E; 264 Amsl) during the post-rainy seasons of 2010/11 and 2011/12. The design comprised 16 incomplete blocks, each made up of 15 plots with three replicates. Sowing was staggered to ensure that all the lines – whether early, medium, or late – are exposed to stress at the same stage of their growth. WS was induced at the flowering stage and continued until grain filling by withholding irrigation, which was resumed in all the maturity groups at the end of the stress treatment. The following observations were recorded for all the 240 lines: anthesis-to-silking interval (ASI, in days), ear girth (EG, in centimetres), ear length (EL, in centimetres), the number of kernels per row (KR), the number of kernel rows (KRN), 100-kernel weight (HKW, in grams), and grain yield (GY, kilograms per plot).

### SNP genotyping

All the 240 genotypes were genotyped with Infinium Maize SNP50 BeadChip (Illumina, San Diego, California, USA) containing 56 110 SNPs published earlier by Nepolean *et al* [26]. A set of 29 619 high-quality SNPs [26] was used for association analysis.

### Data analysis

#### Field data

Mixed model analysis using restricted maximum likelihood approach was performed to estimate the best linear unbiased predictors (BLUPs) of genotypes at each location. Plot-level data from each location were analyzed using the following linear mixed-effects model:


where *y*_*ijk*_ is the observed value of the *k*^th^ genotype in the *j*^th^ incomplete block within the *i*^th^ replication, *μ* is the grand mean, *r*_*i*_ the main effect of the *i*^th^ replication, *r*(*b*)_*ij*_ the nested effect of the *j*^th^ incomplete block in *i*^th^ replication, *g*_*k*_ is the main effect of the *k*^th^ genotype, and *ϵ*_*ijk*_ is the error term of each *y*_*ijk*_ with *N*(*0,σ*^*2*^)*.* At each location, the nested blocks and genotype effects were treated as random and replicated as fixed effects. The residual diagnostic plots indicated that the data satisfied the assumption of normality. To understand the effect of genotypes across locations in greater detail, the data across the three locations were analyzed using the model


where *l*_*m*_ is the main effect of *m*^th^ location and (*gl*)_*mk*_ is the interaction effect of *k*^th^ genotype in *m*^th^ location. Residual variances of individual locations were modelled in combined analysis using a mixed model procedure. BLUPs for the genotypes were also estimated across combined locations. Data were analyzed using a proc *mixed* procedure of the software package SAS ver. 9.3 for Windows (SAS Institute Inc.) [59].

#### Genome-wide association analysis

Two models, Q_10_ + K + Admixture from GenABEL and Q_10_ + K + Admixture from GAPIT were used to identify significant SNPs from BLUPs. Here, admixture values were obtained from earlier data for the same panel [26]. In GenABEL, a mixed linear model (MLM) was employed with polygenic (maximum likelihood method) [60] and “mmscore” functions [61]. In the present study, the K estimate represents the identity by state (IBS) shared by individuals computing to IBS or kinship matrix generated from GenABEL [60]. The population structure was corrected for the first ten components (Q_10_) [12, 62] by implementing the “cmdscale” function in GenABEL [68]. *P* values with 1 degree of freedom were corrected at 5% FDR using the “qvaluebh95” function [63] in GenABEL. In GAPIT, GWAS was investigated by the compressed mixed linear model (CMLM) approach with “EMMAx” (Efficient Mixed Model Association-eXpedited) [64], and *P* values were adjusted at 5% FDR to determine significant associations [64]. To compute kinship matrix, the VanRaden algorithm [65] was implemented. Admixture and principal component analysis (PCA) were used to correct the population structure.

## Availability of supporting data

The raw SNP data (Submission # 10.6070/H4BG2KX8) has been submitted to the website: http://www.labarchives.com/. All the supporting data are included as additional files (Additional files 1, 2, 3, 4, 5, 6, 7, 8, 9, 10, 11).

## Electronic supplementary material

Additional file 1: Table S1: Agronomic performance of genotypes in well-watered and water-stressed conditions. (XLSX 10 KB)

Additional file 2: Table S2: Phenotypic correlation coefficients for agronomic traits in well-watered and water-stressed conditions. Significant phenotypic correlations (*p* < 0.01) are marked with an asterisk. (XLSX 10 KB)

Additional file 3: Table S3: GWA signals observed for seven agronomic traits under water stress. Significant associations (*P* < 5.8 × 10^−4^ as the cut-off) for all agronomic traits were observed at all the three locations (New Delhi, Hyderabad, and Karimnagar) and for the mean of all phenotypic data and four models (M1, M2, M3, and M4) (Refer Methods for details). All FDR-corrected SNP associations are in bold. The drought-tolerant gene annotations (refer Figure 4) are marked with the respective references. (XLSX 48 KB)

Additional file 4: Figure S1: Manhattan plots showing SNPs significantly associated (log_10_ *P* < −4.0) with seven agronomic traits under well-watered conditions measured across M2 (see Methods for details). (TIFF 643 KB)

Additional file 5: Figure S2: Manhattan plots showing SNPs significantly associated (log_10_ *P* < −4.0) to seven agronomic traits under well-watered conditions measured across M1 (see Methods for details). (TIFF 634 KB)

Additional file 6: Figure S3: Manhattan plots showing SNPs significantly associated (log_10_ *P* < −4.0) to seven agronomic traits under well-watered conditions measured across M3 (see Methods for details). (TIFF 635 KB)

Additional file 7: Figure S4: Manhattan plots showing SNPs significantly associated (log_10_ *P* < −4.0) to seven agronomic traits under well-watered conditions measured across M4 (see Methods for details). (TIFF 638 KB)

Additional file 8: Figure S5: Manhattan plots showing SNPs significantly associated (log_10_ *P* < −4.0) to seven agronomic traits under water stress measured across M1 (see Methods for details). (TIFF 634 KB)

Additional file 9: Figure S6: Manhattan plots showing SNPs significantly associated (log_10_ *P* < −4.0) to seven agronomic traits under water stress measured across M3 (see Methods for details). (TIFF 633 KB)

Additional file 10: Figure S7: Manhattan plots showing SNPs significantly associated (log_10_ *P* < −4.0) to seven agronomic traits under water stress measured across M4 (see Methods for details). (TIFF 626 KB)

Additional file 11: Figure S8: Manhattan plots showing SNPs significantly associated (log_10_ *P* < −4.0) with seven agronomic traits under water stress measured across M2 (see Methods for details). (TIFF 628 KB)

## Abbreviations

ABA: Abscisic acid
bHLH: Basic helix-loop-helix
BLUP: Best linear unbiased predictors
CAMTA: Calmodulin binding transcription activator
DCM: DNA-cytosine-5-methyltransferase
ERF: Ethylene responsive factor
FDR: False-discovery-rate
G3PDH: Glycerol-3-phosphate dehydrogenase
GP: Glutathione peroxidase
GST: Glutathione-*S*-transferase
GWA: Genome-wide association
LD: Linkage disequilibrium
MLM: Mixed linear model
NCED: 9-cis-epoxycarotenoid dioxygenase
NF-YA: Nuclear factor-YA
PLD: Phospholipase D
QTL: Quantitative trait locus
ROS: Reactive oxygen species
SBP: Squamous promoter binding protein
SNP: Single nucleotide polymorphism
ZF-HD: Zinc finger-homeodomain.
