# Supplementary figures and images for: Functional mechanisms of drought tolerance in subtropical maize (Zea mays L.) identified using genome-wide association mapping
Source: BMC Genomics. 2014 Dec 24;15(1):1182. doi: 10.1186/1471-2164-15-1182 (PMC4367829; doi:10.1186/1471-2164-15-1182)

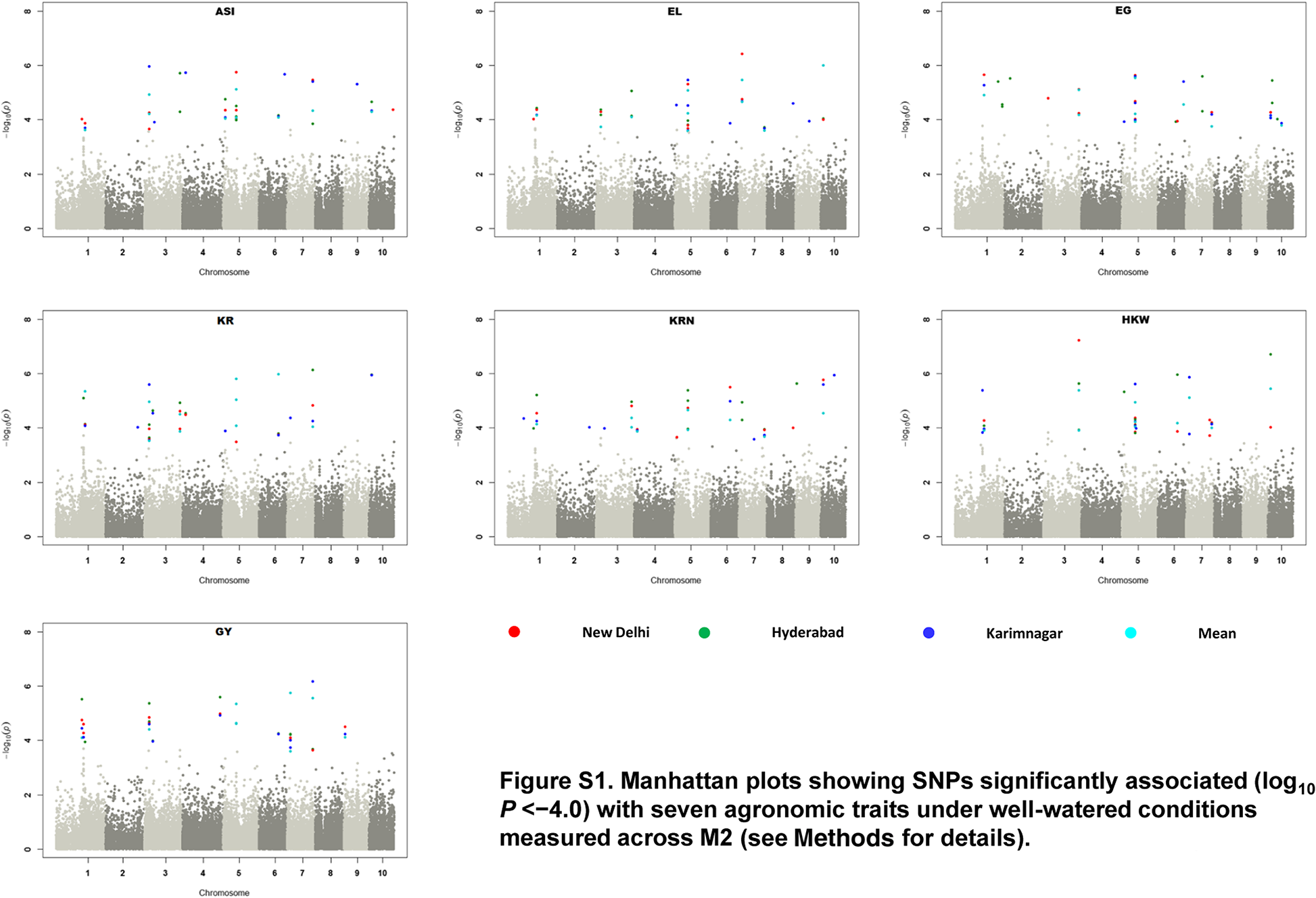

Supplement: Supplementary file 4 — Additional file 4: Figure S1: Manhattan plots showing SNPs significantly associated (log10 P < −4.0) with seven agronomic traits under well-watered conditions measured across M2 (see Methods for details). (TIFF 643 KB) [file 12864_2014_6931_MOESM4_ESM.tiff]

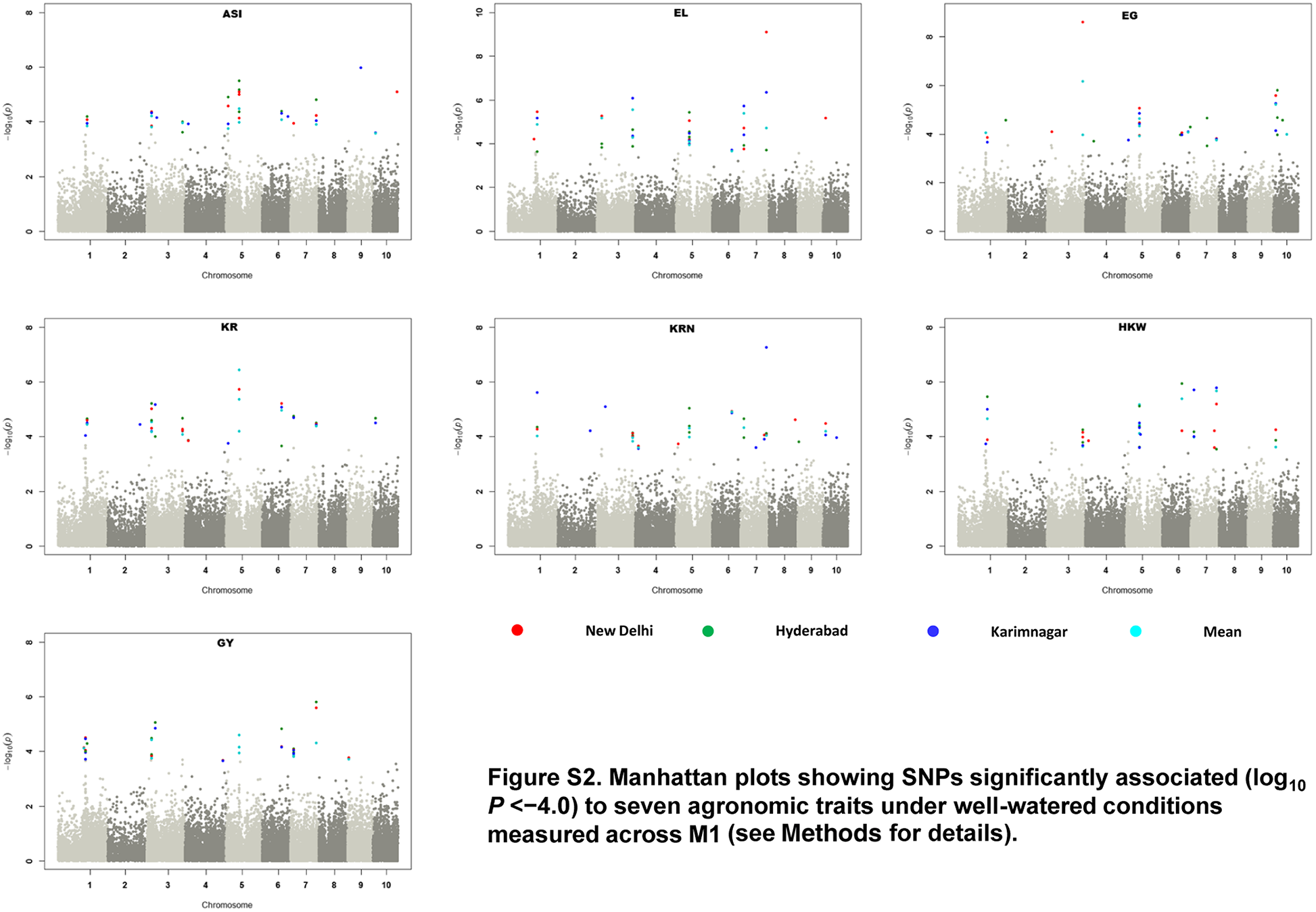

Supplement: Supplementary file 5 — Additional file 5: Figure S2: Manhattan plots showing SNPs significantly associated (log10 P < −4.0) to seven agronomic traits under well-watered conditions measured across M1 (see Methods for details). (TIFF 634 KB) [file 12864_2014_6931_MOESM5_ESM.tiff]

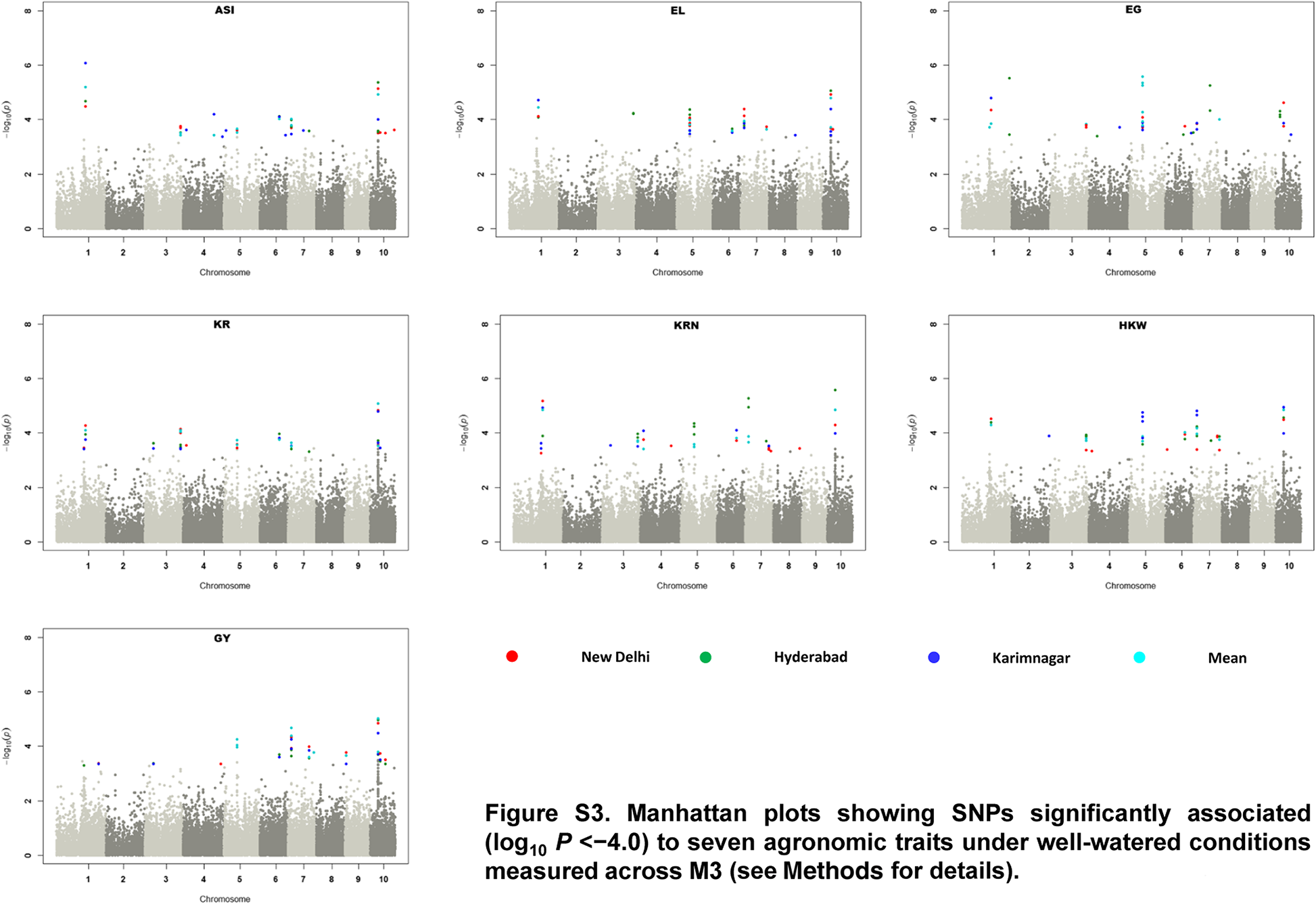

Supplement: Supplementary file 6 — Additional file 6: Figure S3: Manhattan plots showing SNPs significantly associated (log10 P < −4.0) to seven agronomic traits under well-watered conditions measured across M3 (see Methods for details). (TIFF 635 KB) [file 12864_2014_6931_MOESM6_ESM.tiff]

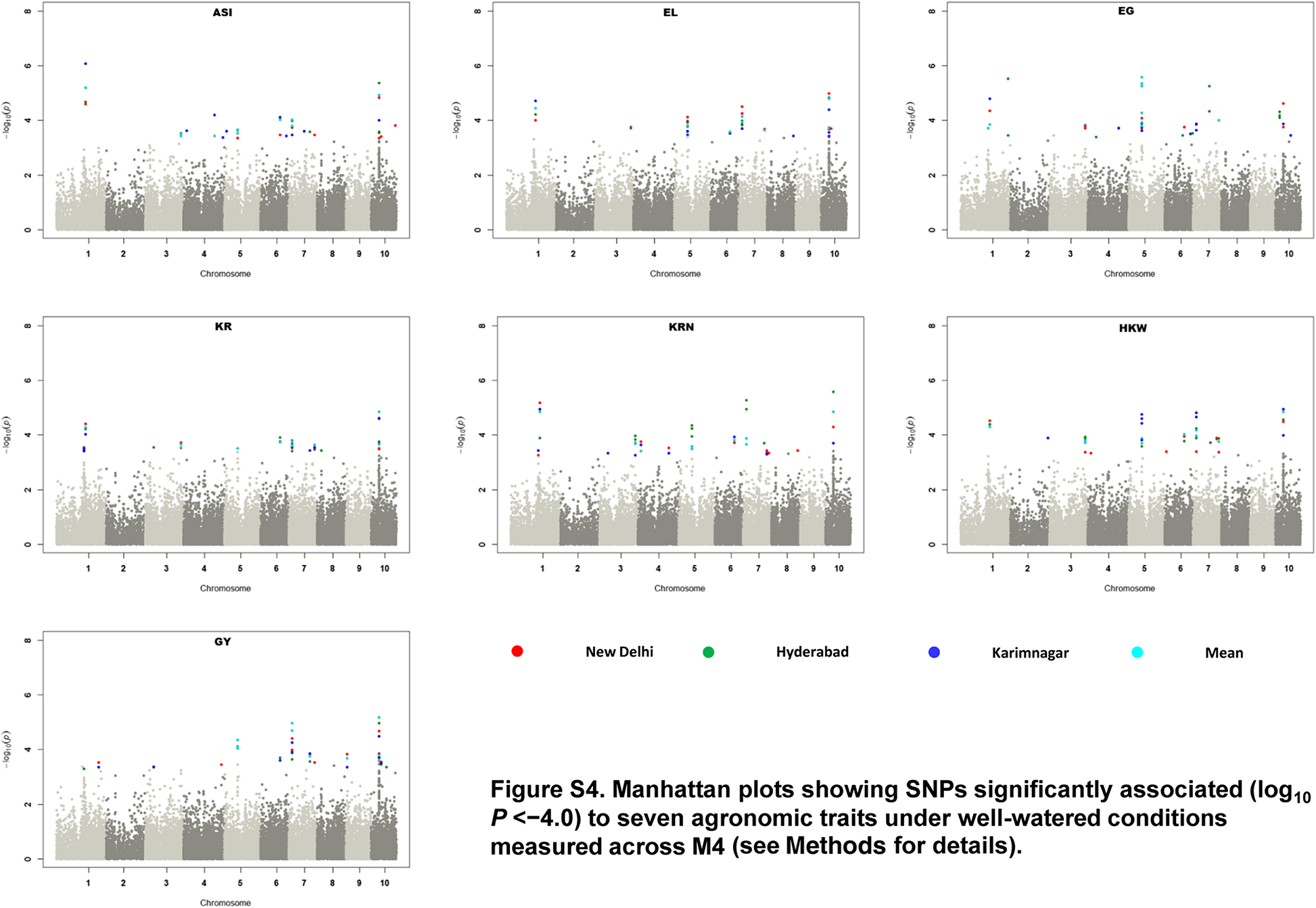

Supplement: Supplementary file 7 — Additional file 7: Figure S4: Manhattan plots showing SNPs significantly associated (log10 P < −4.0) to seven agronomic traits under well-watered conditions measured across M4 (see Methods for details). (TIFF 638 KB) [file 12864_2014_6931_MOESM7_ESM.tiff]

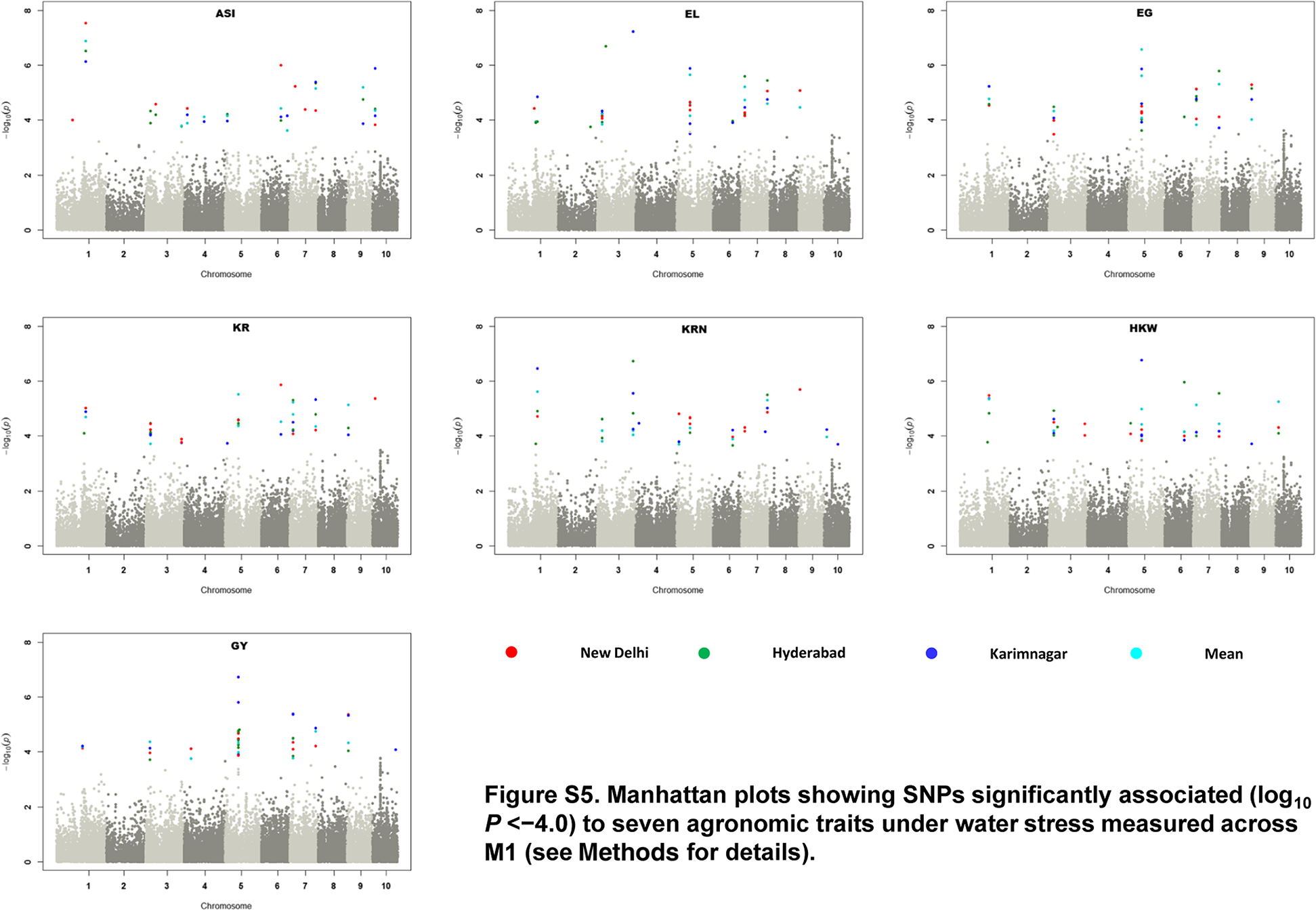

Supplement: Supplementary file 8 — Additional file 8: Figure S5: Manhattan plots showing SNPs significantly associated (log10 P < −4.0) to seven agronomic traits under water stress measured across M1 (see Methods for details). (TIFF 634 KB) [file 12864_2014_6931_MOESM8_ESM.tiff]

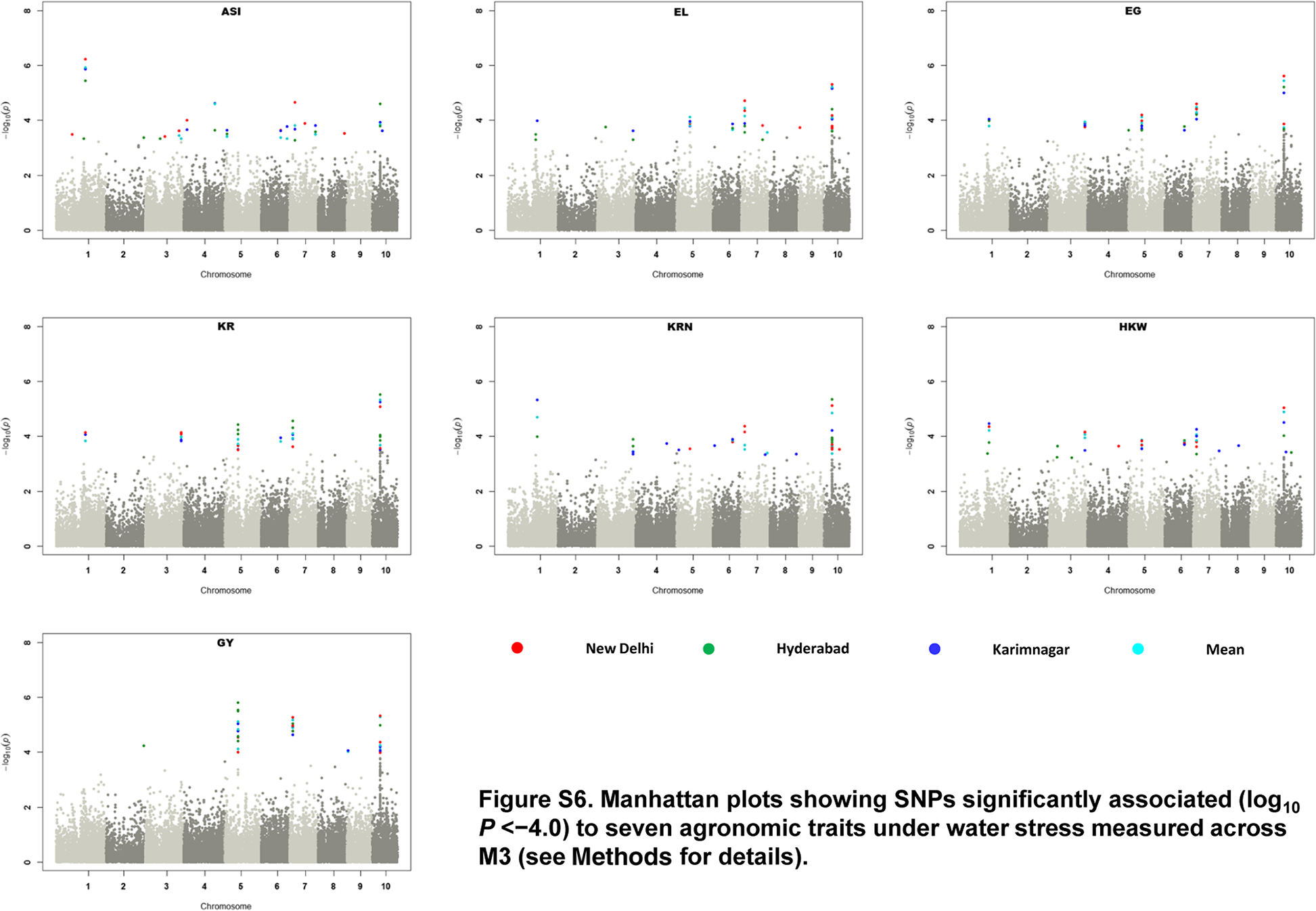

Supplement: Supplementary file 9 — Additional file 9: Figure S6: Manhattan plots showing SNPs significantly associated (log10 P < −4.0) to seven agronomic traits under water stress measured across M3 (see Methods for details). (TIFF 633 KB) [file 12864_2014_6931_MOESM9_ESM.tiff]

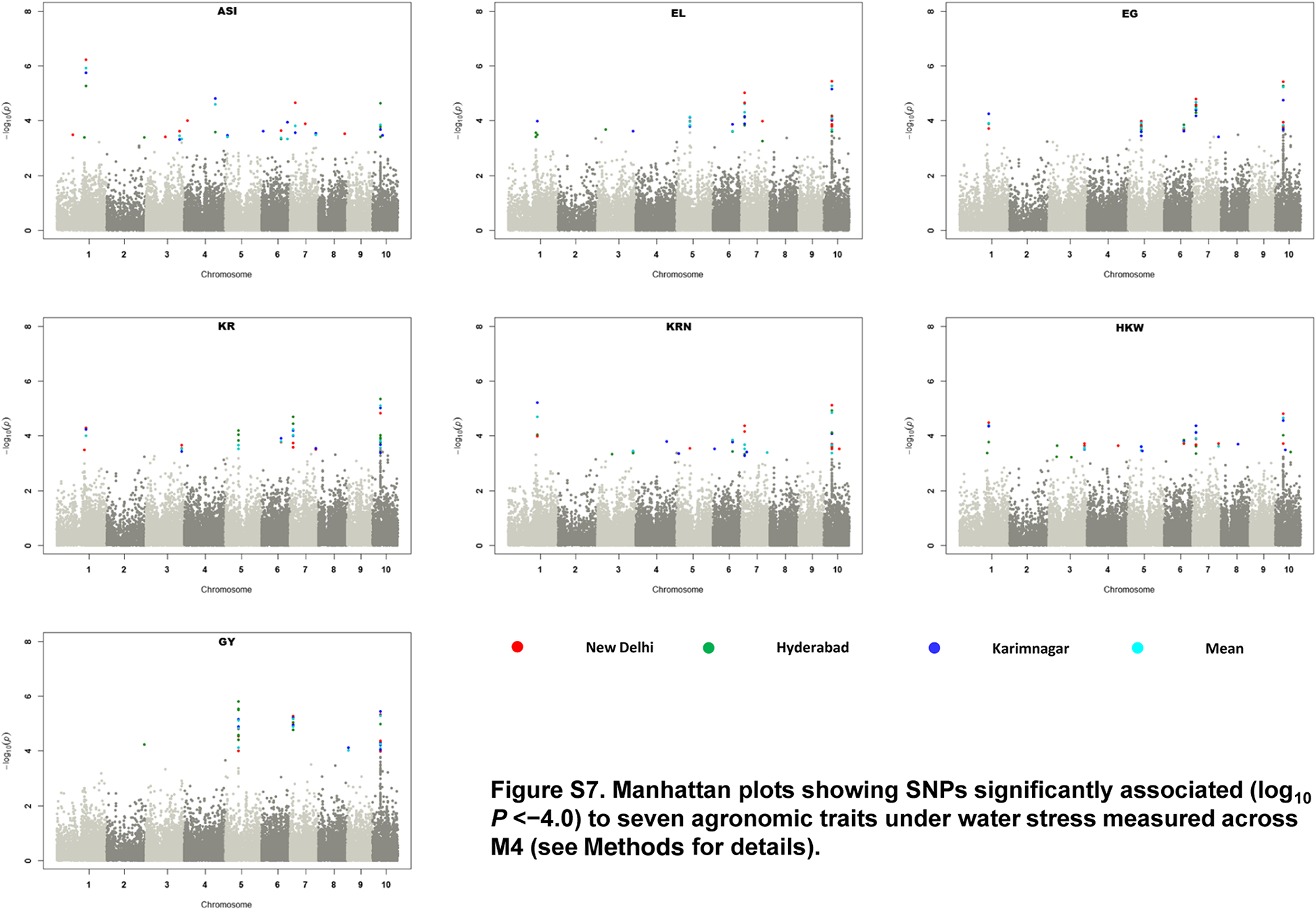

Supplement: Supplementary file 10 — Additional file 10: Figure S7: Manhattan plots showing SNPs significantly associated (log10 P < −4.0) to seven agronomic traits under water stress measured across M4 (see Methods for details). (TIFF 626 KB) [file 12864_2014_6931_MOESM10_ESM.tiff]

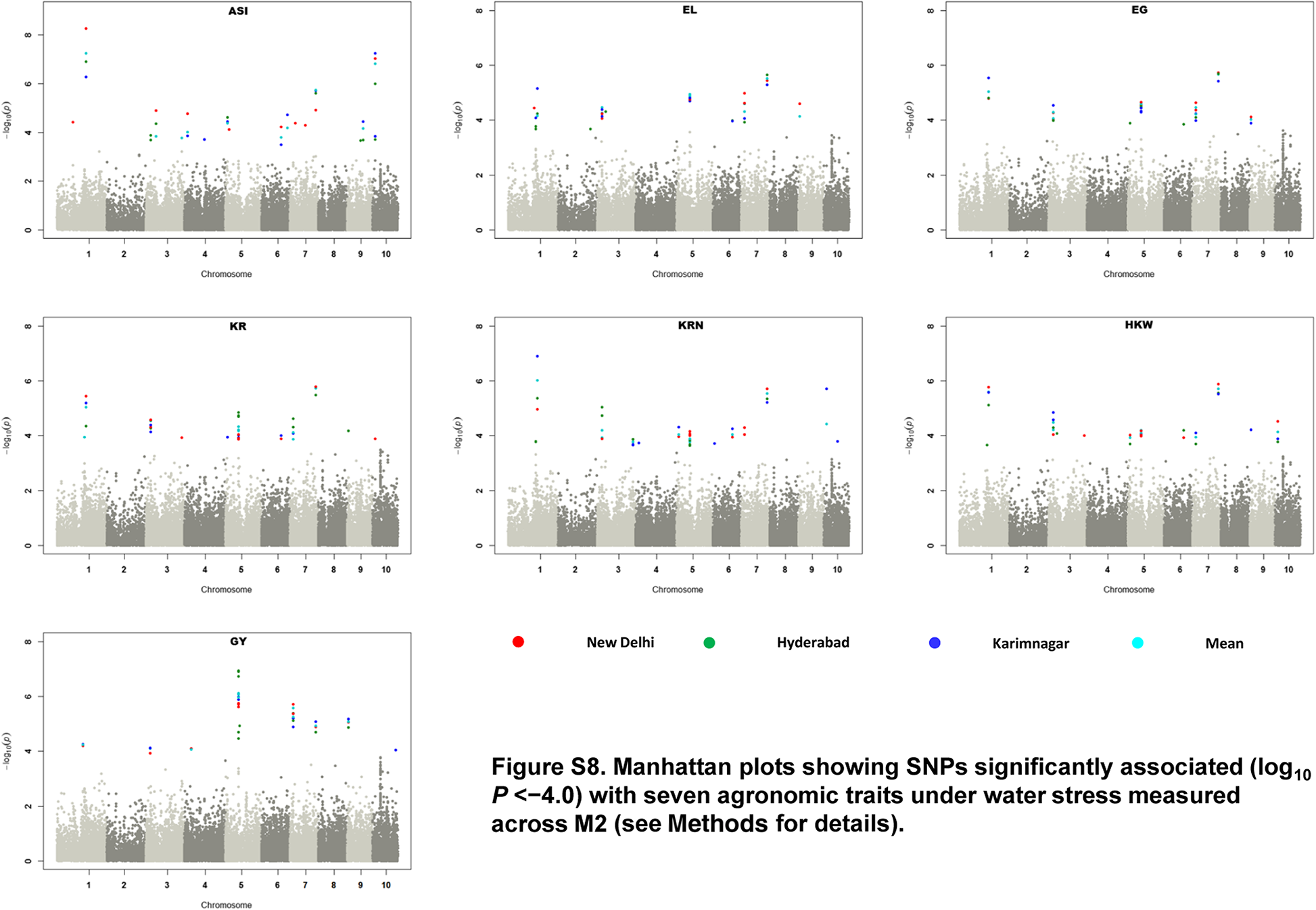

Supplement: Supplementary file 11 — Additional file 11: Figure S8: Manhattan plots showing SNPs significantly associated (log10 P < −4.0) with seven agronomic traits under water stress measured across M2 (see Methods for details). (TIFF 628 KB) [file 12864_2014_6931_MOESM11_ESM.tiff]
